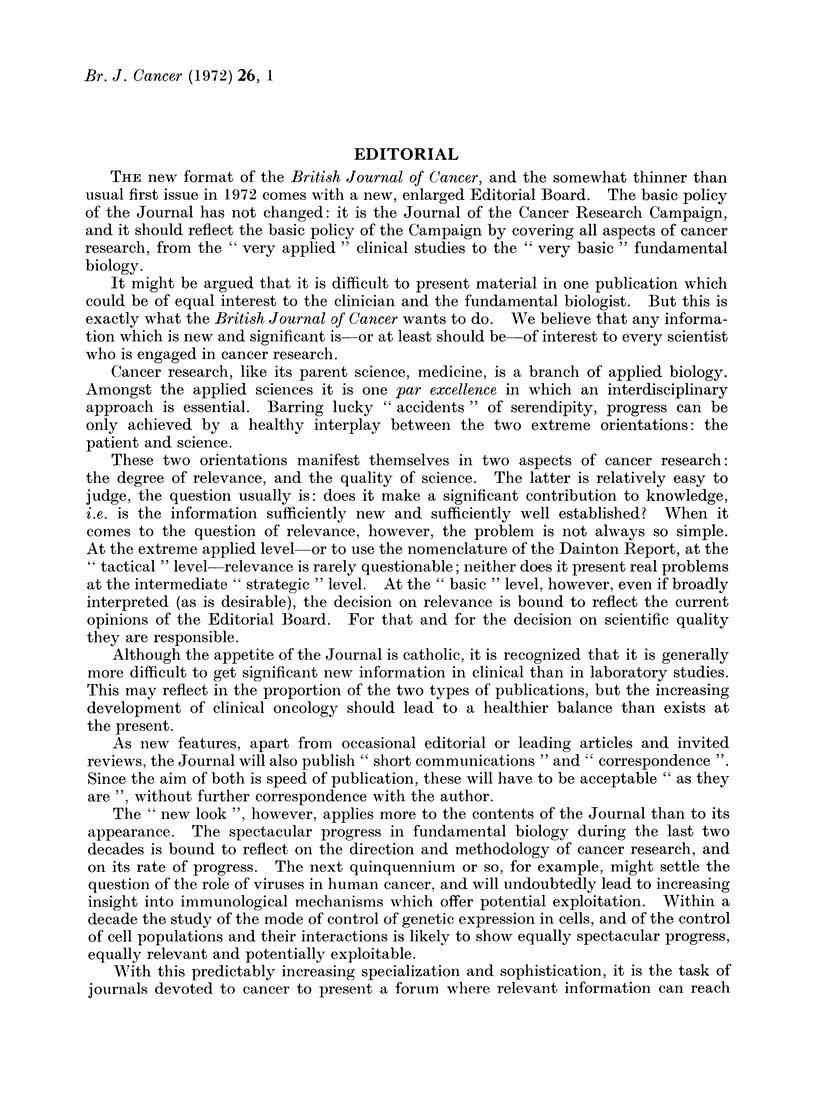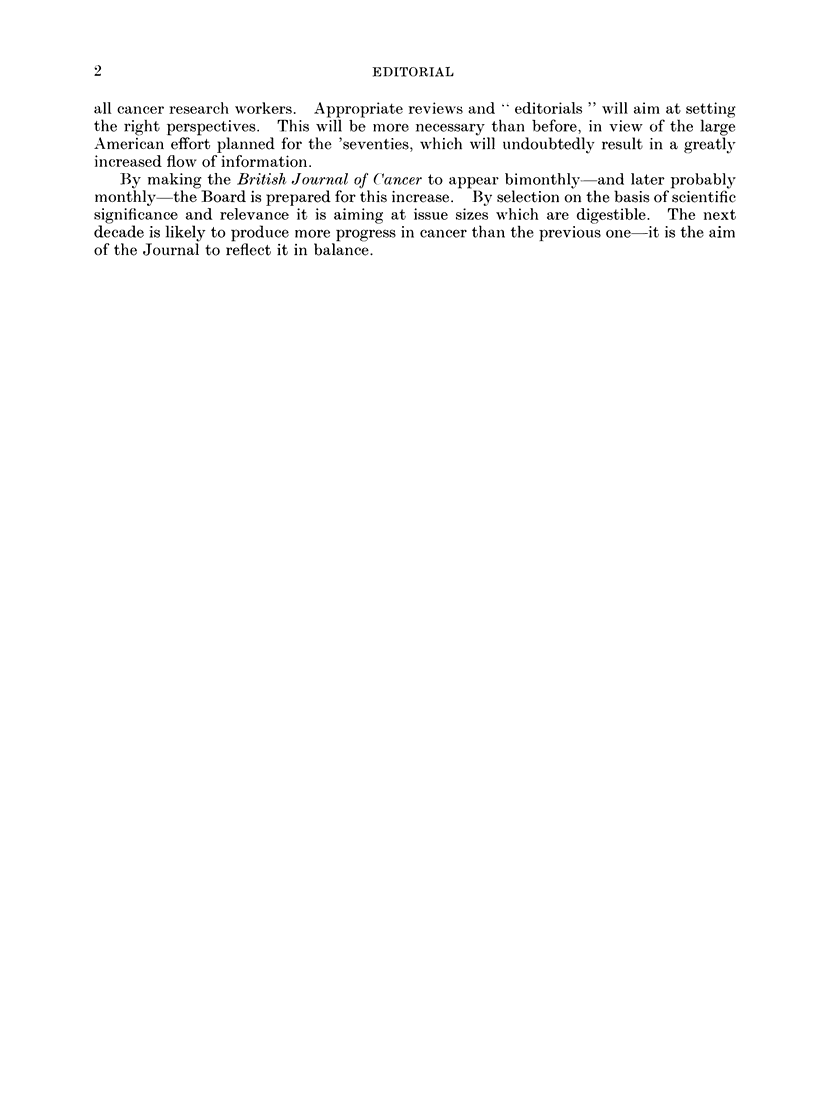# Editorial

**Published:** 1972-02

**Authors:** 


*Br. J. Cancer* (1972) **26**, 1

### EDITORIAL

THE new format of the *British Journal of Cancer*, and the somewhat thinner than usual first issue in 1972 comes with a new, enlarged Editorial Board. The basic policy of the Journal has not changed: it is the Journal of the Cancer Research Campaign, and it should reflect the basic policy of the Campaign by covering all aspects of cancer research, from the " very applied " clinical studies to the " very basic " fundamental biology.

It might be argued that it is difficult to present material in one publication which could be of equal interest to the clinician and the fundamental biologist. But this is exactly what the *British Journal of Cancer* wants to do. We believe that any information which is new and significant is—or at least should be—of interest to every scientist who is engaged in cancer research.

Cancer research, like its parent science, medicine, is a branch of applied biology. Amongst the applied sciences it is one *par excellence* in which an interdisciplinary approach is essential. Barring lucky " accidents " of serendipity, progress can be only achieved by a healthy interplay between the two extreme orientations: the patient and science.

These two orientations manifest themselves in two aspects of cancer research: the degree of relevance, and the quality of science. The latter is relatively easy to judge, the question usually is: does it make a significant contribution to knowledge, *i.e.* is the information sufficiently new and sufficiently well established? When it comes to the question of relevance, however, the problem is not always so simple. At the extreme applied level—or to use the nomenclature of the Dainton Report, at the " tactical " level—relevance is rarely questionable; neither does it present real problems at the intermediate " strategic " level. At the " basic " level, however, even if broadly interpreted (as is desirable), the decision on relevance is bound to reflect the current opinions of the Editorial Board. For that and for the decision on scientific quality they are responsible.

Although the appetite of the Journal is catholic, it is recognized that it is generally more difficult to get significant new information in clinical than in laboratory studies. This may reflect in the proportion of the two types of publications, but the increasing development of clinical oncology should lead to a healthier balance than exists at the present.

As new features, apart from occasional editorial or leading articles and invited reviews, the Journal will also publish " short communications " and " correspondence ". Since the aim of both is speed of publication, these will have to be acceptable " as they are ", without further correspondence with the author.

The " new look ", however, applies more to the contents of the Journal than to its appearance. The spectacular progress in fundamental biology during the last two decades is bound to reflect on the direction and methodology of cancer research, and on its rate of progress. The next quinquennium or so, for example, might settle the question of the role of viruses in human cancer, and will undoubtedly lead to increasing insight into immunological mechanisms which offer potential exploitation. Within a decade the study of the mode of control of genetic expression in cells, and of the control of cell populations and their interactions is likely to show equally spectacular progress, equally relevant and potentially exploitable.

With this predictably increasing specialization and sophistication, it is the task of journals devoted to cancer to present a forum where relevant information can reach all cancer research workers. Appropriate reviews and " editorials " will aim at setting the right perspectives. This will be more necessary than before, in view of the large American effort planned for the 'seventies, which will undoubtedly result in a greatly increased flow of information.

By making the *British Journal of Cancer* to appear bimonthly—and later probably monthly—the Board is prepared for this increase. By selection on the basis of scientific significance and relevance it is aiming at issue sizes which are digestible. The next decade is likely to produce more progress in cancer than the previous one—it is the aim of the Journal to reflect it in balance.